# Orthorexic tendency and its association with weight control methods and dietary variety in Polish adults: a cross-sectional study

**DOI:** 10.3389/fnut.2024.1355871

**Published:** 2024-04-22

**Authors:** Marta Plichta, Joanna Kowalkowska

**Affiliations:** ^1^Department of Food Market and Consumer Research, Institute of Human Nutrition Sciences, Warsaw University of Life Sciences (SGGW-WULS), Warsaw, Poland; ^2^Department of Human Nutrition, Faculty of Food Science, University of Warmia and Mazury in Olsztyn, Olsztyn, Poland

**Keywords:** orthorexic tendency, weight control methods, dietary variety, adults, Poland

## Abstract

The methods for controlling weight play a central role in formally diagnosed eating disorders (EDs) and appear to be important in the context of other nonformally recognized disorders, such as orthorexia nervosa (ON). These methods also have an impact on eating behaviors, including dietary variety. Our study aimed to: (i) assess the intensity of ON tendency by sex and BMI groups, (ii) evaluate the associations between ON tendency, weight control methods, and dietary variety, and (iii) determine the extent to which weight control methods and dietary variety contribute to the ON tendency among both females and males. Data were gathered from a sample of 936 Polish adults (463 females and 473 males) through a cross-sectional quantitative study conducted in 2019. Participants were requested to complete the ORTO-6, the Weight Control Methods Scale, and the Food Intake Variety Questionnaire (FIVeQ). Multiple linear regression analysis was employed to evaluate associations between ON tendency, weight control methods, and dietary variety. Females exhibited a higher ON tendency than males (14.4 ± 3.4 vs. 13.5 ± 3.7, *p* < 0.001, *d* = 0.25). In the regression model, the higher ON tendency was predicted by more frequent use of weight control methods, such as restricting the amount of food consumed, using laxatives, and physical exercise among both females and males as well as following a starvation diet in females, and drinking teas to aid bowel movements among males. Moreover, the higher ON tendency was predicted by higher dietary variety, lower age in both sexes, and higher level of education among males. However, there were no differences in ON tendency across BMI groups. In conclusion, the findings showed that ON tendency was predicted by a higher frequency of weight control methods commonly used by individuals with anorexia nervosa (AN) and bulimia nervosa (BN). The resemblance to these two EDs is also suggested by the higher intensity of ON tendency among females and younger people. However, the prediction of ON tendency by dietary variety indicates that the obsessive preoccupation with healthy eating may not be advanced enough to observe a decrease in the dietary variety among these individuals.

## 1 Introduction

Orthorexia nervosa (ON) is defined as an obsessive preoccupation with healthy eating (1). It involves adhering to strict dietary rules that prioritize food quality and purity, potentially resulting in the exclusion of specific foods or food categories deemed “harmful to health” (2). Individuals with a tendency toward ON experience intense stress, anxiety, shame, heightened fear, feelings of personal impurity, and negative physical sensations when unable to strictly follow the perceived healthy dietary rules (3). This tendency can lead to interpersonal, academic, or social impairment as well as substantial weight loss, nutritional deficiencies, and other medical consequences (2).

Until now, ON has not been formally classified as a distinct mental disorder in classifications like the DSM-5 (4) or the ICD-11 (5). This lack of categorization seems to stem from previous difficulties in establishing a consistent definition of ON, its status as an independent disorder, and the standardized criteria for its classification and diagnosis (6). Though several authors have proposed diagnostic criteria for ON, it has still been difficult to establish any standard or consensus (6). Only recently such an attempt has been made to consolidate current knowledge about ON and to develop consistent terminology and defining characteristics of ON (7). Nevertheless, the recognition of ON as a separate mental disease is impeded by the perceived overlap between ON’s characteristics and those of eating disorders, particularly anorexia nervosa (AN) (8). Researchers propose that ON might exist on the same spectrum as AN and bulimia nervosa (BN), with individuals transitioning from an obsession with food quantity to an emphasis on its quality (9). Moreover, they suggest that a focus on a healthy diet could be a socially acceptable method of weight control for those with AN and BN (10). In contrast, other researchers highlight distinct differences between ON, AN, and BN (11–13).

Originally, there was a belief in the literature that individuals with ON tendency were not focused on weight loss and did not exhibit negative attitudes toward their body image (14). In contrast, individuals with AN and BN have a self-concept that centers on appearance, leading them to control their diet to influence body shape and weight (15). Mixed results have been found to support this perspective regarding a potential relationship between body mass index (BMI) and ON tendency (11, 16–20). In AN, however, BMI is a critical criterion for determining disease severity (4, 5). While the definition of ON recognizes health preoccupation rather than appearance preoccupation as the basis of ON psychopathology, the documented association with the drive for thinness among females suggests that preoccupation with weight and shape may still be present (21). Studies indeed indicate that females with ON tendency, especially, experience poor body image and self-esteem (18, 22, 23). Similarly, a study involving fitness participants with ON tendency observed that these individuals internalized the ideal of thinness, experiencing social physique anxiety related to body image dissatisfaction and eating disorders (EDs) (24). Additionally, a panel of experts recently concluded that if an individual’s drive to lose weight is related to health beliefs and/or anxiety, it is consistent with ON (7). However, if an individual’s drive to lose weight is related to body dissatisfaction, dysphoria, and/or body dysmorphia, it may be more consistent with AN. Body image is associated with the perception of physical appearance, satisfaction levels, and behaviors individuals engage in or avoid due to discomfort with their bodies (3). Therefore, people with EDs may employ various methods of weight control to attain and/or maintain a thin body and weight, including restrictive diets, starvation, purging behaviors, and others (4, 5). Hence, it is worthwhile to investigate whether there is an association between ON tendency and weight control methods. Only one study assessed substance use for weight control among individuals with ON tendency, focusing exclusively on smoking, drinking alcohol, and using illicit drugs (25).

Importantly, literature has demonstrated that a restrictive diet may heighten the risk of EDs (26). Several factors associated with AN and BN may also be associated with ON tendency, such as an excessive focus on food, strict adherence to dietary rules, and food restriction (27). Furthermore, researchers have emphasized that ON tendency may escalate with the adoption of more restrictive eating behaviors (28), suggesting a less diverse diet among individuals with ON tendency. Once again, only one study has examined ON tendency and dietary variety (29). However, the researchers presented a mean score of dietary variety for the total sample, and when focusing on ON tendency they only considered the consumption of selected food groups (29). Therefore, our study aimed: (i) to assess the intensity of ON tendency by sex and BMI groups; (ii) to evaluate the associations between ON tendency, weight control methods, and dietary variety; and (iii) to determine the extent to which weight control methods and dietary variety contribute to ON tendency among both females and males. To the best of our knowledge, no study has yet explored these aspects. In addition, conducting studies on ON tendency in Poland is particularly important because it can help to better understand this phenomenon and obtain a more complete view of it in the Polish population. The following research questions were formulated: (1) Do sex and BMI determine the intensity of ON tendency, the frequency of weight control methods, and dietary variety? (2) Does an increase in the intensity of ON tendency lead to a higher frequency of weight control methods? (3) Does an increase in the intensity of ON tendency result in less dietary variety? We also have put forward the following hypotheses:

*H1*: Females, compared to males, exhibit a higher intensity of ON tendency, a higher frequency of using weight control methods, and more dietary variety.

*H2*: BMI determines the frequency of weight control methods and dietary variety, but it does not determine ON tendency.

*H3*: People with a higher intensity of ON tendency are characterized by a higher frequency of weight control methods.

*H4*: The diet of people with a higher intensity of ON tendency is characterized by a lower variety.

## 2 Materials and methods

### 2.1 Study design and participant recruitment

**Figure 1:**
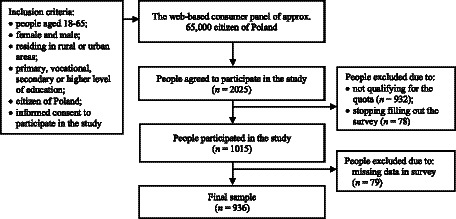
Study design and participant recruitment.

A cross-sectional quantitative survey was conducted from February to March 2019. Participant recruitment and data collection were undertaken by the professional market research agency ARC Market and Opinion in Warsaw, Poland (30). Data were gathered using the computer-assisted web interviewing technique (CAWI). Approximately 65,000 people from all over Poland have registered for the web-based consumer panel. In the study, quota sampling was applied to ensure a representative sample of the population of adult Poles, considering sociodemographic characteristics such as sex, age, place of residence, and education level. Upon receiving an invitation to participate, 2025 people aged 18–65 expressed their agreement to take part in the study. During the recruitment process, 932 individuals did not meet the quota criteria, and 78 individuals discontinued survey completion. Throughout the collection process, the agency was responsible for ensuring that the proportions of selected characteristics in the sample were consistent with the proportions of those characteristics in the general population. Supplementary Figure S1 shows a detailed description of the quota sampling of adult Poles in the study. Participants were not obliged to answer sensitive questions to avoid making them uncomfortable. Due to missing data, 79 participants were excluded by researchers from the database. Ultimately, the study included 936 participants (Figure 1).

### 2.2 Sociodemographic characteristics

Sociodemographic data were gathered through self-reports, utilizing both open and closed questions. These included inquiries about sex (“female,” “male”), age (in years), current weight (in kg), height (in cm), place of residence (“village,” “city with 100,000 citizens or less,” “city with more than 100,000 citizens”), and education level (“primary or vocational,” “secondary,” “higher”). The BMI was computed as the ratio of an individual’s weight (in kg) to the square of their height (in meters) (31). Subsequently, participants’ BMI values were categorized into four groups following the World Health Organization guidelines (31): underweight (BMI <18.5 kg/m^2^), normal weight (BMI between 18.5 and 24.9 kg/m^2^), pre-obesity (BMI between 25.0 and 29.9 kg/m^2^), and obesity (BMI ≥30.0 kg/m^2^).

### 2.3 Orthorexic tendency

The assessment of orthorexic tendency utilized the Polish version of the ORTO-6 questionnaire (32). The ORTO-6 is a shortened version of the original ORTO-15 questionnaire, which was adapted and validated in a group of adult females and males in Poland (33). The ORTO-6 was developed based on estimates from meta-analysis and Monte Carlo simulations considering five studies using ORTO questionnaires (32), including a Polish validation study of ORTO-15 (33). The ORTO-6 is a self-report, multiple-choice questionnaire comprising six items (e.g., “Do you think that the conviction to eat only healthy food increases self-esteem?”), measured on a four-point Likert scale. To enhance interpretability, the coding method has been reversed compared to the ORTO-6 questionnaire. Items 3, 4, 7, 10, 11, and 12 were scored as follows: 1—"never,” 2—"sometimes,” 3—"often,” and 4—"always.” The total score was derived by summing the six items, resulting in a score range of 6 to 24 points. A higher score on this scale indicates a higher intensity of orthorexic tendency. In the present study, McDonald’s Omega for the ORTO-6 was 0.751 in the total sample, 0.713 for females, and 0.777 for males.

### 2.4 Dietary variety

Dietary variety was evaluated using the Food Intake Variety Questionnaire (FIVeQ), which is a self-report and semi-qualitative food intake frequency questionnaire validated in Poles (34). The FIVeQ captures the frequency of consumption of 63 food subgroups over the past 7 days. The food list of the FIVeQ encompasses eight major food groups: (1) cereal products, (2) fruit, (3) vegetables, (4) dairy products, (5) meat products, (6) fats, (7) sugar and sweets, and (8) beverages. Participants indicated whether they consumed more than the suggested amounts of various food products, considering them as already prepared “ready to eat,” i.e., indicating the amount “eaten off the plate” (e.g., “Have you eaten more than about 2 tablespoons of the following types of fruits in the past 7 days?”). Responses were recorded on a dichotomous scale: “yes” or “no.” If participants did not remember whether they had consumed a particular food product, they selected the “no” answer. Quantities for foods were specified, for instance, as: “seven slices of whole bread or roll” for wholemeal cereal products, “two tablespoons” for large grains groats, “seven glasses” for milk and dairy products, “amount for 1 slice of bread, well covered” for sausages, “two items” for all kinds of eggs, “10 cubes of chocolate” for various chocolate types, “one wine glass (100 mL)” for wine, and “one vodka glass (50 mL)” for vodka and other strong alcohols. The complete list of food products and their quantities is provided in the source publication (34). In the present study, McDonald’s Omega for the FIVeQ was 0.908 in the total sample, 0.901 for females, and 0.913 for males.

Dietary variety was quantified using the Food Intake Variety Index (FIVeI), calculated as the sum of consumed food subgroups during the week, excluding alcohols (beer, wine, and vodka). The maximum FIVeI is 60 food products per week. Participants were categorized into one of four groups based on their FIVeI, reflecting different levels of dietary variety: (1) inadequate (<20 food products per week), (2) sufficient (20–29 food products per week), (3) good (30–39 food products per week), and (4) very good (≥40 food products per week).

### 2.5 Weight control methods

The frequency of weight control methods was measured by the Weight Control Methods Scale (35). This tool was developed and validated among both Polish females and males, with an average age of 34.1 ± 13.9 years. It is a self-report tool comprises a single question (i.e., “How often do you use the following weight control methods?”) addressing eight methods of weight control: (1) restricting the amount of food consumed, (2) starvation diet, (3) using appetite suppressant pills, (4) provoking vomiting, (5) using laxatives, (6) drinking teas to aid bowel movements, (7) physical exercise, and (8) smoking cigarettes. Respondents indicated the frequency of weight control methods on a nine-point scale, where: 1—"I never used,” 5—"it is hard to say,” and 9—"I use regularly.” The possible range of scores for each method was from 1 to 9, with a higher score indicating more frequent use of a particular method of weight control. In the present study, McDonald’s Omega for the Weight Control Methods Scale was 0.710 in the total sample, 0.686 for females, and 0.730 for males.

### 2.6 Statistical analysis

Categorical variables were expressed as percentages (%), while continuous variables were presented as means with standard deviation (SD). The normality of the distribution for continuous variables was examined through a normal probability plot. The homogeneity of variance was evaluated using Levene’s test and the Brown–Forsythe test.

The associations between independent categorical variables were established using the Pearson chi-squared test. Differences in mean values for two independent samples with a normal distribution were analyzed using Student’s *t*-test, while for those with an abnormal distribution, the U-Mann Whitney test was employed. The differences between the mean values for multiple independent samples with a normal distribution were examined through one-way analysis of variance (ANOVA) and *post hoc* Tukey’s test. For abnormal distribution, the Kruskal-Wallis Rank ANOVA test was utilized, followed by multiple comparisons of mean ranks for all samples. The associations between continuous variables were explored using Pearson’s correlation coefficient for normal distribution and Kendall’s tau correlation coefficient for abnormal distribution.

The associations between orthorexic tendency and weight control methods as well as dietary variety were assessed separately for females and males, through multiple linear regression analysis adjusting for age (continuous; in years), place of residence (categorical; village/city with 100,000 citizens or less/city with more than 100,000 citizens), education level (categorical; primary or vocational/secondary/higher), and BMI (continuous; kg/m^2^). Before conducting the analysis, scatter plots were created to confirm the linearity of variables. Due to the absence of linearity, data normalization was performed using a logarithmic transformation. Orthorexic tendency, treated as a continuous variable was introduced into the models as the dependent variable. The independent variables included in the models were weight control methods (continuous; points) and dietary variety (continuous; food products/week). Stepwise forward selection of variables was utilized in the models. Standardized regression coefficients (β) and unstandardized regression coefficients (B) with a 95% confidence interval (CI) were employed. The analysis conducted on both models explained 25% of the variance for model 1 including females (*R*^2^ = 0.25) and 29% of the variance for model 2 including males (*R*^2^ = 0.29). The Durbin–Watson statistic for model 1 was 2.02 and for model 2 was 2.06, indicating that the assumption of no correlation of the random components was satisfied. The assumption of no correlation between predictors was evaluated using a collinearity test to calculate the Tolerance Factor and Variance Inflation Factor (VIF). For both model 1 and model 2, the VIF did not exceed 10.0, while the Tolerance Factor was higher than 0.1, which confirms the assumption that the predictors are not correlated (36). For all tests, a *p*-value <0.05 was considered significant. Statistical analyses were performed using Statistica software, version 13.3 PL (StatSoft Inc., Tulsa, OK, USA; StatSoft, Krakow, Poland), and IBM SPSS Statistics for Windows, version 29.0 (IBM Corp, Armonk, NY, USA).

The G*Power software version 3.1.9.7 was used to calculate the effect sizes of analyses (Heinrich-Heine-Universität Düsseldorf, Düsseldorf, Germany). Different statistical tests have different effect sizes, therefore we matched the appropriate effect sizes for the tests used. For the Student’s *t*-test, we applied Cohen’s d coefficient; for the Whitney’s *U*-Mann test—Glass’s rank bivariate correlation coefficient; for the Pearson chi-squared test—Cramer’s V coefficient; for the one-way analysis of variance (ANOVA)—eta squared; and for the Kruskal-Wallis Rank ANOVA—epsilon squared.

## 3 Results

### 3.1 Sample characteristics

The study sample comprised 936 Polish adults, with 49.5% being females (Table 1). The mean age of the respondents was 41.4 ± 13.8 years. Females were younger than males (*p* = 0.006; *d* = 0.15). A majority of the participants resided in cities (63.8%). The highest proportion of participants had a primary or vocational level of education (38.8%). The mean BMI was 25.6 ± 4.7 kg/m^2^. Males had a higher BMI compared to females (*p* < 0.001; *r*_g_ = 0.30).

**Table 1:** Sociodemographic characteristics of the study sample across sex groups.

Variables	Total sample	Female	Male	U/t/Chi^2^	Df	V/d/r_g_	*p*-value
	*N* = 936	*N* = 463	*N* = 473				
Age in years (M ± SD)	41.4 ± 13.8	40.2 ± 14.2	42.6 ± 13.3	−2.71^●●^	934	0.15^▲▲^	**0.006**
Age in categories *n* (%)							
18–24	113 (12.1)	67 (14.5)	46 (9.7)	24.67^●●●^	4	0.16^▲^	**<0.001**
25–34	218 (23.3)	120 (25.9)	98 (20.7)				
35–44	216 (23.1)	104 (22.5)	112 (23.7)				
45–54	169 (18.0)	57 (12.3)	112 (23.7)				
55–65	220 (23.5)	115 (24.8)	105 (22.2)				
Place of residence *n* (%)							
Village	339 (36.2)	181 (39.1)	158 (33.4)	3.38^●●●^	2	0.06^▲^	0.184
City ≤100,000 citizens	305 (32.6)	146 (31.5)	159 (33.6)				
City >100,000 citizens	292 (31.2)	136 (29.4)	156 (32.9)				
Education level *n* (%)							
Primary or vocational	363 (38.8)	163 (35.2)	200 (42.3)	5.09^●●●^	2	0.07^▲^	0.078
Secondary	326 (34.8)	173 (37.4)	153 (32.3)				
Higher	247 (26.4)	127 (27.4)	120 (25.4)				
BMI in kg/m^2^ (M ± SD)	25.6 ± 4.7	24.9 ± 5.1	26.3 ± 4.2	−5.67^●^	891	0.30^▲▲▲^	**<0.001**
BMI categories *n* (%)							
Underweight	38 (4.1)	30 (6.5)	8 (1.7)	29.93^●●●^	3	0.18^▲^	**<0.001**
Normal weight	424 (45.3)	234 (50.5)	190 (40.2)				
Pre-obesity	321 (34.3)	131 (28.3)	190 (40.2)				
Obesity	153 (16.3)	68 (14.7)	85 (17.9)				

^●^ U-Mann Whitney test (U); ^●●^ Student’s *t*-test (t); ^●●●^ Pearson chi-squared test (Chi^2^); ^▲^ Cramer’s V coefficient (V); ^▲▲^ Cohen’s d coefficient (d); ^▲▲▲^ Glass’s rank bivariate correlation coefficient (r_g_); N, number of participants; %, sample percentage; M, mean; SD, standard deviation; Df, degrees of freedom; BMI, body mass index. Bold values indicate statistical significance.

### 3.2 Differences in orthorexic tendency, weight control methods, and dietary variety across sex and BMI

The mean ORTO-6 score, indicating the ON tendency, was 13.9 ± 3.6. Females exhibited a higher ON tendency than males (*p* < 0.001; *d* = 0.25; Table 2). Physical exercise (5.5 ± 2.3) and restricting the amount of food consumed (4.6 ± 2.3) were the most frequently used methods of weight control, while provoking vomiting was the least frequently used (1.6 ± 1.6). In comparison to males, females more frequently utilized weight control methods such as restricting the amount of food consumed (*p* < 0.001; *d* = 0.36), using appetite suppressant pills (*p* = 0.017; *r*_g_ = 0.17), using laxatives (*p* = 0.028; *r*_g_ = 0.16), and drinking teas to aid bowel movements (*p* < 0.001; *r*_g_ = 0.12). A majority of individuals exhibited good dietary variety (36.6%). No significant differences in the mean FIVeI as well as the percentage distribution in the dietary variety categories were observed across sex groups.

**Table 2:** Orthorexic tendency, weight control methods, and dietary variety in the total sample and sex groups.

Variables	Total sample	Female	Male	U/t/Chi^2^	Df	V/d/r_g_	*p*-value
	*N* = 936	*N* = 463	*N* = 473				
Orthorexic tendency (M ± SD)	13.9 ± 3.6	14.4 ± 3.4	13.5 ± 3.7	3.89^●●^	934	0.25^▲▲^	**<0.001**
Weight control methods (M ± SD)							
Restricting the amount of food consumed	4.6 ± 2.3	5.0 ± 2.3	4.2 ± 2.2	5.15^●●^	934	0.36^▲▲^	**<0.001**
Starvation diet	2.3 ± 1.9	2.4 ± 2.0	2.2 ± 1.9	0.82^●^	891	0.10^▲▲▲^	0.411
Using appetite suppressant pills	1.9 ± 1.8	2.0 ± 1.9	1.7 ± 1.6	2.37^●^	891	0.17^▲▲▲^	**0.017**
Provoking vomiting	1.6 ± 1.6	1.5 ± 1.5	1.6 ± 1.6	−1.38^●^	891	0.06^▲▲▲^	0.168
Using laxatives	1.9 ± 1.9	2.1 ± 1.9	1.8 ± 1.8	2.20^●^	891	0.16^▲▲▲^	**0.028**
Drinking teas to aid bowel movements	3.0 ± 2.5	3.3 ± 2.6	2.6 ± 2.2	4.07^●^	891	0.12^▲▲▲^	**<0.001**
Physical exercise	5.5 ± 2.3	5.6 ± 2.1	5.5 ± 2.4	0.628^●^	891	0.04^▲▲▲^	0.529
Smoking cigarettes	3.6 ± 3.0	3.4 ± 3.0	3.7 ± 2.9	−1.23^●●^	934	0.10^▲▲^	0.216
Dietary Variety *n* (%)							
Inadequate	90 (9.6)	47 (10.2)	43 (9.1)	0.33^●●●^	3	0.02^▲^	0.952
Sufficient	286 (30.6)	140 (30.2)	146 (30.9)				
Good	343 (36.6)	170 (36.7)	173 (36.6)				
Very good	217 (23.2)	106 (22.9)	111 (23.5)				
FIVeI (M ± SD)	32.5 ± 10.7	32.2 ± 10.5	32.8 ± 11.0	−0.84^●●^	934	0.06^▲▲^	0.397

^●^ U-Mann Whitney test (U); ^●●^ Student’s *t*-test (t); ^●●●^ Pearson chi-squared test (Chi^2^); ^▲^ Cramer’s V coefficient (V); ^▲▲^ Cohen’s d coefficient (d); ^▲▲▲^ Glass’s rank bivariate correlation coefficient (r_g_); M, mean; SD, standard deviation; Df, degrees of freedom; FIVeI, food intake variety index. Bold values indicate statistical significance.

Females with obesity were more likely to employ weight control method such as restricting the amount of food consumed compared to those with underweight, normal weight, and pre-obesity, while females with normal weight and those with pre-obesity were more likely to employ this method that females with underweight (*p* < 0.001; *ε*^2^ = 0.30; Table 3). The use of starvation diet was more prevalent among females with obesity compared to those with underweight (*p* = 0.004; *ε*^2^ = 0.17). Additionally, females with obesity more frequently use appetite suppressant pills (*p* < 0.001; *ε*^2^ = 0.25) and laxatives (*p* < 0.001; *ε*^2^ = 0.21) than females with underweight and those with normal weight. Females with underweight less frequently drank teas to aid bowel movements than those with other BMI groups (*p* < 0.001; *ε*^2^ = 0.20). No significant differences were observed in ON tendency, provocation of vomiting, physical exercise, cigarette smoking, and dietary variety measured by the score (FIVeI) and in categories across the BMI groups.

**Table 3:** Orthorexic tendency, weight control methods, and dietary variety in females across the BMI groups.

Variables	Underweight	Normal weight	Pre-obesity	Obesity	H/F/Chi^2^	V/η^2^/ε^2^	Df	*p*-value
	*N* = 30	*N* = 234	*N* = 131	*N* = 68				
Orthorexic tendency (M ± SD)	14.2 ± 3.9	14.4 ± 3.6	14.4 ± 3.2	14.4 ± 3.2	0.03^●●^	0.01^▲▲^	3	0.989
Weight control methods (M ± SD)								
Restricting the amount of food consumed	3.0 ± 2.6 ^a^	4.9 ± 2.4 ^b,c^	5.1 ± 2.1 ^b,c^	5.9 ± 1.7 ^d^	30.51^●^	0.30^▲▲▲^	3	**<0.001**
Starvation diet	1.6 ± 1.4 ^a,b,c^	2.2 ± 1.9 ^a,b,c,d^	2.4 ± 1.9 ^a,b,c,d^	3.0 ± 2.2 ^b,c,d^	13.49^●^	0.17^▲▲▲^	3	**0.004**
Using appetite suppressant pills	1.1 ± 0.7 ^a,b,c^	1.8 ± 1.9 ^a,b,c^	2.2 ± 1.9 ^a,b,c,d^	3.0 ± 2.4 ^c,d^	38.79^●^	0.25^▲▲▲^	3	**<0.001**
Provoking vomiting	1.2 ± 0.7	1.5 ± 1.5	1.6 ± 1.7	1.7 ± 1.6	1.03^●●^	0.07^▲▲^	3	0.377
Using laxatives	1.3 ± 1.5 ^a,b,c^	1.9 ± 1.8 ^a,b,c^	2.3 ± 2.0 ^a,b,c,d^	2.8 ± 2.3 ^c,d^	23.23^●^	0.21^▲▲▲^	3	**<0.001**
Drinking teas to aid bowel movements	1.8 ± 2.0 ^a^	3.1 ± 2.6 ^b,c,d^	3.5 ± 2.6 ^b,c,d^	4.1 ± 2.8 ^b,c,d^	19.74^●^	0.20^▲▲▲^	3	**<0.001**
Physical exercise	5.2 ± 2.4	5.8 ± 2.3	5.5 ± 2.0	5.4 ± 1.7	5.86^●^	0.09^▲▲▲^	3	0.118
Smoking cigarettes	4.2 ± 3.5	3.3 ± 2.9	3.5 ± 3.1	3.6 ± 3.1	0.83^●●^	0.07^▲▲^	3	0.478
Dietary Variety *n* (%)								
Inadequate	7 (23.3)	22 (9.4)	11 (8.4)	7 (10.3)	12.54^●●●^	0.09^▲^	9	0.184
Sufficient	9 (30.0)	76 (32.5)	39 (29.8)	16 (23.5)				
Good	12 (40.0)	78 (33.3)	51 (38.9)	29 (42.7)				
Very good	2 (6.7)	58 (24.8)	30 (22.9)	16 (23.5)				
FIVeI (M ± SD)	27.6 ± 8.9	32.6 ± 10.7	32.2 ± 10.4	32.5 ± 10.0	2.04^●●^	0.11^▲▲^	3	0.107

^●^ Kruskal-Wallis Rank ANOVA (H); ^●●^ One-way analysis of variance (ANOVA) (F); ^●●●^ Pearson chi-squared test (Chi^2^); ^a,b,c,d^ the means differ statistically significantly at *p* < 0.05; ^▲^ Cramer’s V coefficient (V); ^▲▲^ eta squared (η^2^); ^▲▲▲^ epsilon squared (ε^2^); M, mean; SD, standard deviation; Df, degrees of freedom; FIVeI, food intake variety index. Bold values indicate statistical significance.

Males with obesity and pre-obesity were more likely to employ method such as restricting the amount of food consumed compared to those with underweight and normal weight (*p* < 0.001; *ε*^2^ = 0.30; Table 4). Physical exercise was more commonly practiced by males with normal weight and pre-obesity compared to those with obesity (*p* = 0.009; *η*^2^ = 0.16). Males with obesity more frequently smoke cigarettes than those with pre-obesity (*p* = 0.028; *η*^2^ = 0.14). Moreover, among males, significant differences in dietary variety measured in categories across BMI groups were identified (*p* = 0.017; *V* = 0.12). The highest percentage of males with underweight had inadequate dietary variety (50.0%), while those with normal weight, pre-obesity, and obesity had good dietary variety (36.2, 38.9, and 34.1%, respectively). No significant differences were found in ON tendency, other weight control methods, and FIVeI across BMI groups.

**Table 4:** Orthorexic tendency, weight control methods, and dietary variety in males across the BMI groups.

Variables	Underweight	Normal weight	Pre-obesity	Obesity	H/F/Chi^2^	V/η^2^/ε^2^	Df	*p*-value
	*N* = 8	*N* = 190	*N* = 190	*N* = 85				
Orthorexic tendency (M ± SD)	13.8 ± 5.9	13.6 ± 3.8	13.8 ± 3.5	12.6 ± 3.3	2.24^●●^	0.11^▲▲^	3	0.083
Weight control methods (M ± SD)								
Restricting the amount of food consumed	2.0 ± 1.5 ^a,b^	3.7 ± 2.4 ^a,b^	4.6 ± 2.0 ^c,d^	4.9 ± 1.9 ^c,d^	33.69^●^	0.30^▲▲▲^	3	**<0.001**
Starvation diet	2.0 ± 2.1	2.2 ± 2.0	2.3 ± 1.9	2.3 ± 1.9	0.11^●●^	0.03^▲▲^	3	0.954
Using appetite suppressant pills	1.6 ± 1.4	1.7 ± 1.6	1.7 ± 1.5	1.8 ± 1.7	0.21^●●^	0.03^▲▲^	3	0.892
Provoking vomiting	1.6 ± 1.4	1.7 ± 1.7	1.6 ± 1.5	1.5 ± 1.4	0.34^●●^	0.05^▲▲^	3	0.793
Using laxatives	2.0 ± 1.5	1.9 ± 1.9	1.8 ± 1.8	1.7 ± 1.4	0.39^●●^	0.05^▲▲^	3	0.756
Drinking teas to aid bowel movements	2.4 ± 1.7	2.5 ± 2.3	2.8 ± 2.4	2.4 ± 1.9	0.79^●●^	0.09^▲▲^	3	0.504
Physical exercise	4.8 ± 2.9 ^a,b,c,d^	5.7 ± 2.6 ^a,b,c^	5.6 ± 2.2 ^a,b,c^	4.7 ± 2.3 ^a,d^	3.89^●●^	0.16^▲▲^	3	**0.009**
Smoking cigarettes	2.9 ± 2.7 ^a,b,c,d^	3.7 ± 3.0 ^a,b,c,d^	3.4 ± 2.9 ^a,b,c^	4.5 ± 3.0 ^a,b,d^	3.05^●●^	0.14^▲▲^	3	**0.028**
Dietary Variety *n* (%)								
Inadequate	4 (50.0)	16 (8.4)	14 (7.4)	9 (10.6)	20.17^●●●^	0.12^▲^	9	**0.017**
Sufficient	1 (12.5)	63 (33.2)	53 (27.9)	29 (34.1)				
Good	1 (12.5)	69 (36.2)	74 (38.9)	29 (34.1)				
Very good	2 (25.0)	42 (22.1)	49 (25.8)	18 (21.2)				
FIVeI (M ± SD)	29.1 ± 17.6	32.1 ± 10.5	33.6 ± 10.6	32.7 ± 12.3	0.85^●●^	0.08^▲▲^	3	0.468

^●^ Kruskal-Wallis Rank ANOVA (H); ^●●^ One-way analysis of variance (ANOVA) (F); ^●●●^ Pearson chi-squared test (Chi^2^); ^a,b,c,d^ the means differ statistically significantly at *p* < 0.05; ^▲^ Cramer’s V coefficient (V); ^▲▲^ eta squared (η^2^); ^▲▲▲^ epsilon squared (ε^2^); M, mean; SD, standard deviation; Df, degrees of freedom; FIVeI, food intake variety index. Bold values indicate statistical significance.

### 3.3 Prediction of orthorexic tendency by weight control methods and dietary variety, and the associations between them across sex

A positive correlation was identified between ON tendency and various weight control methods, including restricting the amount of food consumed, starvation diet, using appetite suppressant pills, provoking vomiting, using laxatives, drinking teas to aid bowel movements, and physical exercise for both females and males (*p* < 0.001 for all; Table 5). Furthermore, ON tendency showed a positive correlation with FIVeI for both sexes (*p* < 0.001). However, it’s noteworthy that these correlations were weak to moderate.

**Table 5:** Correlations between orthorexic tendency, weight control methods, and dietary variety among females and males.

Variables	1	2	3	4	5	6	7	8	9	10
Orthorexic tendency (1)		0.354 ^r^ ***	0.274 ^r^ ***	0.121 ^τ^ ***	0.134 ^τ^ ***	0.167 ^τ^ ***	0.260 ^r^ ***	0.319 ^r^ ***	−0.023 ^τ^	0.304 ^r^ ***
Restricting the amount of food consumed (2)	0.333 ^r^ ***		0.317 ^r^ ***	0.179 ^τ^ ***	0.058 ^τ^	0.171 ^τ^ ***	0.357 ^r^ ***	0.344 ^r^ ***	0.044 ^τ^	0.090 ^r^
Starvation diet (3)	0.289 ^r^ ***	0.335 ^r^ ***		0.321 ^τ^ ***	0.399 ^τ^ ***	0.353 ^τ^ ***	0.378 ^r^ ***	0.098 ^r^ *	0.131 ^τ^ ***	0.157 ^r^ **
Using appetite suppressant pills (4)	0.249 ^τ^ ***	0.188 ^τ^ ***	0.492 ^τ^ ***		0.381 ^τ^ ***	0.448 ^τ^ ***	0.325 ^τ^ ***	0.001 ^τ^	0.172 ^τ^ ***	0.109 ^τ^ **
Provoking vomiting (5)	0.187 ^τ^ ***	0.111 ^τ^ **	0.456 ^τ^ ***	0.628 ^τ^ ***		0.396 ^τ^ ***	0.204 ^τ^ ***	0.001 ^τ^	0.171 ^τ^ ***	0.089 ^τ^ *
Using laxatives (6)	0.245 ^τ^ ***	0.157 ^τ^ ***	0.457 ^τ^ ***	0.607 ^τ^ ***	0.600 ^τ^ ***		0.441 ^τ^ ***	0.024 ^τ^	0.083 ^τ^ *	0.166 ^τ^ ***
Drinking teas to aid bowel movements (7)	0.388 ^r^ ***	0.288 ^r^ ***	0.435 ^r^ ***	0.481 ^τ^ ***	0.395 ^τ^ ***	0.512 ^τ^ ***		0.212 ^r^ ***	0.094 ^τ^ *	0.187 ^r^ ***
Physical exercise (8)	0.319 ^r^ ***	0.352 ^r^ ***	0.148 ^r^ *	0.030 ^τ^	0.006 ^τ^	0.062 ^τ^ *	0.179 ^r^ ***		−0.063 ^τ^ *	0.153 ^r^ **
Smoking cigarette (9)	0.024 ^r^	−0.013 ^r^	0.108 ^r^ *	0.190 ^τ^ ***	0.196 ^τ^ ***	0.158 ^τ^ ***	0.151 ^r^ *	−0.006 ^r^		0.021 ^τ^
FIVeI (10)	0.294 ^r^ ***	0.071 ^r^	0.187 ^r^ ***	0.154 ^τ^ ***	0.157 ^τ^ ***	0.187 ^τ^ ***	0.279 ^r^ ***	0.160 ^r^ **	0.153 ^r^ **	

Correlations for females are presented above the main diagonal, while correlations for males are presented below the main diagonal; *p < 0.05; **p < 0.001; ***p < 0.0001; r, Pearson’s correlation coefficient; τ, tau Kendall’s correlation coefficient; FIVeI, food intake variety index.

An increase in the intensity of ON tendency was predicted by a higher FIVeI, and more frequent use of weight control methods, including restricting the amount of food consumed, using laxatives, and physical exercise among both females and males (Tables 6, 7). Moreover, among females higher ON tendency was predicted by more frequent use of a starvation diet as a weight control method, while among males by drinking teas to aid bowel movements. Considering sociodemographic characteristics, an increase in the intensity of ON tendency was predicted by younger age among both sexes and higher level of education among males.

**Table 6:** Multiple linear regression analysis of orthorexic tendency in females group.

Parameter	Adjusted Model						
	*B*	SE	*β*	95% CI	*F*	Df	*p*-value
Age	−0.08	0.03	−0.13	−0.14; −0.03	9.49	1	**0.002**
Restricting the amount of food consumed	0.08	0.02	0.21	0.04; 0.12	21.31	1	**<0.001**
Starvation diet	0.03	0.02	0.09	0.00; 0.06	3.98	1	**0.046**
Using laxatives	0.03	0.02	0.09	0.00; 0.06	4.18	1	**0.041**
Physical exercise	0.09	0.02	0.20	0.05; 0.13	20.62	1	**<0.001**
FIVeI	0.11	0.03	0.19	0.0; 0.17	19.74	1	**<0.001**

B, unstandardized regression coefficient; β, standardized regression coefficient; 95% CI, 95% confidence interval; (ref.), reference group; the model adjusted for: age (continuous; in years), place of residence (categorical; village/city ≤ 100,000 citizens/city > 100,000 citizens), education level (categorical; primary or vocational/secondary/higher), and BMI (continuous; kg/m^2^); FIVeI, food intake variety index. Bold values indicate statistical significance.

**Table 7:** Multiple linear regression analysis of orthorexic tendency in males group.

Parameter	Adjusted Model						
	*B*	SE	*β*	95% CI	*F*	Df	*p*-value
Age	−0.15	0.03	−0.17	−0.21; −0.08	19.18	1	**<0.001**
Primary/vocational education (ref. higher)	−0.05	0.02	−0.13	−0.07; −0.02	4.69	2	**0.002**
Restricting the amount of food consumed	0.07	0.02	0.19	0.04; 0.12	19.49	1	**<0.001**
Using laxatives	0.05	0.02	0.11	0.01; 0.09	5.48	1	**0.019**
Drinking teas to aid bowel movements	0.07	0.02	0.19	0.04; 0.11	15.91	1	**<0.001**
Physical exercise	0.06	0.02	0.14	0.03; 0.09	10.62	1	**0.001**
FIVeI	0.07	0.03	0.12	0.03; 0.13	8.65	1	**0.003**

B, unstandardized regression coefficient; β, standardized regression coefficient; 95% CI, 95% confidence interval; (ref.), reference group; the model adjusted for: age (continuous; in years), place of residence (categorical; village/city ≤ 100,000 citizens/city > 100,000 citizens), education level (categorical; primary or vocational/secondary/higher), and BMI (continuous; kg/m^2^); FIVeI, food intake variety index. Bold values indicate statistical significance.

## 4 Discussion

In this study, we investigated the association between ON tendency and the frequency of utilizing various weight control methods and dietary variety, as well as examined the extent to which the methods of weight control and dietary variety contribute to ON tendency in females and males. Previous studies suggested that ON was not associated with a disturbed self-image, fear of obesity, or any inclination toward thinness (37, 38). Its initial focus was on food quality driven by health concerns (14). Therefore, it seemed improbable to resort to weight control methods deemed inappropriate or harmful to health (39). However, in our study, a positive association between ON tendency and such methods as restricting the amount of food consumed, following a starvation diet, using appetite suppressant pills, provoking vomiting, using laxatives, and drinking teas to aid bowel movements was found among both females and males. These methods are typically associated with individuals experiencing eating disorders such as AN and BN (4, 5). Notably, these methods constitute formal diagnostic criteria for both AN and BN (4, 5). Methods of self-controlling body weight, including the restricting and binge-eating/purging types, are also characteristic of AN (4, 5). This finding underscores the substantial similarity between ON, AN, and BN, extending to behaviors that were traditionally considered unlikely in the context of ON (37). It’s worth noting that individuals with AN and/or BN also exhibit disturbances in body weight and/or body shape perception, an excessive influence of body weight and/or body shape on self-esteem, and a fear of weight gain (4, 5). Until recently, these factors were considered crucial in distinguishing ON from AN and BN (37, 38). Although our study did not include a body image assessment, other studies have demonstrated an association between ON tendency and a drive for thinness, low body satisfaction, low body acceptance, high preoccupation with overweight, or high appearance orientation (18, 40–42).

Excessive exercise is a prevalent method of weight control among individuals with AN and BN (4, 5). In our study, we also identified a positive association between ON tendency and physical exercise for both sexes. It was stated that intense and/or frequent exercises may be related to ON (7). Some researchers even emphasize that individuals particularly susceptible to ON tendency are those who are physically active and/or engaged in professional sports (43). Hence, physical activity appears to be a noteworthy aspect in the context of ON tendency. However, based on our study, we can only determine the frequency of physical exercise as a method of weight control, without specifying its intensity. Consequently, it is challenging for us to discern whether the physical exercise undertaken by the subjects was excessively intense or whether it was health-promoting. It is noteworthy that only cigarette smoking did not exhibit any relationship with ON tendency neither among females nor among males. Other researchers have also reported no association between ON tendency and smoking status (21, 44, 45). Perhaps, the subjects perceived the use of stimulants such as cigarette smoking as an excessively unhealthy method of weight control.

Furthermore, the inconsistent findings regarding the association between ON tendency and BMI have been presented as justification for distinguishing ON from AN and BN (18). Numerous studies have reported no significant association between ON tendency and BMI (11–13). In contrast, BMI serves as a pivotal indicator for diagnosing AN and assessing its severity (4, 5). Our study also did not reveal a direct relationship between ON tendency and specific BMI categories or BMI measured as a continuous variable neither among females nor among males. This observation prompts consideration of whether there is a BMI threshold to distinguish whether extreme underweight in ON tendency indicates underlying AN (7).

Interestingly, our study revealed that females exhibited a higher intensity of ON tendency than males, and ON tendency decreased with age for both sexes. These results diverge from previous inconsistent findings, portraying ON as a more cohesive psychological construct, sharing similarities with formally recognized EDs. Similarly, age and sex are also characteristics related to AN and BN (46). Both AN and BN typically onset during puberty or in young adults (47). However, there is a current trend toward a lower age of onset for these disorders (46). These conditions are more prevalent in females than in males (48, 49), although there has been a recent increase in the prevalence of these disorders in males (50, 51). In contrast, studies on ON have yielded inconsistent results regarding its relationship with sex and age (20, 52–54). Some researchers suggested a higher likelihood of ON tendency in females (52), while others indicated the same for males (20). On the other hand, there are studies reporting no association between sex and ON tendency (53). A similar inconsistency was observed in the case of age (54), with some studies showing no association between ON tendency and age (11, 12, 16, 55), and its increase with age (56). In contrast, other studies have demonstrated that a younger age may be a determining factor in the severity of ON tendency (20, 57).

Furthermore, certain researchers have associated the prevalence of ON tendency with a higher knowledge of health and proper nutrition (58, 59), often linked to increased education (60, 61). Individuals acquiring knowledge about health or human nutrition during their studies may actively seek information on health and nutrition independently (62). Consequently, these individuals might be more susceptible to diverse diets, potentially leading to ON tendency (11, 19, 63, 64). However, this association has been reported inconsistently in the literature (54). Some studies have identified an inverse relationship between ON tendency and education (56, 65), while others have found no such correlation (11). Conversely, there are studies reporting a higher prevalence of ON tendency in individuals with higher education (10). This finding was also supported by our study, nevertheless, it was only observed in males.

Essentially, ON is characterized by an obsessive fixation on food quality, involving long-term, meticulous planning, purchasing, and food preparation (14). Individuals with ON tendency are also preoccupied with the methods and materials used in food production (66). They may exclude genetically modified foods, items high in fat, salt, or sugar, and products containing pesticides, herbicides, and other undesirable components from their diets (16). Over time, they progressively eliminate more foods or even entire food groups, and in extreme cases, they may choose not to eat at all rather than consume foods perceived as unhealthy (67, 68). Consequently, it might be anticipated that individuals with ON tend to follow a relatively low-variety diet. The intriguing finding of our study is that the higher intensity of ON tendency was associated with the higher dietary variety among both females and males. Healthy eating and the obsessive preoccupation with healthy eating are positioned at the extreme ends of the continuum scale representing states varying in the intensity of ON tendency (54). Hence, determining the point at which healthy eating transforms into a pathological obsession with healthy eating proves challenging (54). The positive associations discovered between ON tendency and dietary variety, as well as weight control methods, might be attributed to the fact that the behaviors of individuals along the continuum are shifting toward the extreme pole of ON. However, the process may not be advanced enough to discern a decline in dietary variety among adults studied.

### 4.1 Strengths and limitations

A notable strength of our study lies in its nationally representative sample, encompassing diverse demographics such as age, sex, place of residence, and education level. Consequently, the findings can be generalized to the entire Polish population. The study was conducted by qualified interviewers who employed a robust selection process for the study group, enhancing the credibility of the results. However, it is crucial to exercise caution when extrapolating our results to populations from other countries. Additionally, the comparable number of female and male participants enabled a reliable examination of results by sex. The use of the CAWI technique was instrumental in preserving respondent anonymity and facilitating candid responses to sensitive questions (69). Finally, to the best of our knowledge, this study represents the first exploration of the associations between ON tendency, methods of weight control, and dietary variety.

Nonetheless, the study is subject to several limitations. Primarily, potential biases may arise when analyzing self-reported data. The self-reported weight and height could introduce bias into BMI calculated based on them. Moreover, the accuracy of self-reported weight and height may be influenced by sociodemographic characteristics, such as sex (70). In self-reported surveys, males tend to overestimate height, while females tend to underestimate weight. Additionally, the FIVeQ has been validated in the elderly only. However, the validity of self-report dietary assessment tools is generally better in younger than older adults (71, 72), thus it may be assumed that the FIVeQ validity would be even better in young and middle-aged adult Poles than elderly ones. Moreover, it is the only available tool for assessing dietary variety which takes into account the specifics of the Polish diet, including traditional foods/dishes such as fermented products. Next, the cross-sectional design of our study, with data collection at a single point in time, precluded the assessment of causality in associations between variables. Lastly, despite the significance of the multiple linear regression models (*p* < 0.001 for both), it explained only 25% of the variance in the dependent variable for model 1, and 29% for model 2.

## 5 Conclusion

The findings from our study showed positive associations between the ON tendency and frequency of using various weight control methods as well as the dietary variety among adult Poles, both females and males. Females exhibited a higher ON tendency than males and used certain unhealthy methods of weight control more often. A higher ON tendency was predicted by younger age, higher dietary variety, and more frequent use of weight control methods, such as restricting the amount of food consumed, using laxatives, and physical exercise among both females and males as well as following a starvation diet in females, and drinking teas to aid bowel movements among males. Such methods are also commonly used by individuals with AN or BN and could serve as an early indicator of EDs or the presence of symptomatic but undiagnosed EDs. The resemblance to these EDs is also suggested by the higher intensity of ON tendency among females and younger people. However, the positive prediction of ON tendency by dietary variety indicates that the obsessive preoccupation with healthy eating may not be advanced enough to observe a decrease in the dietary variety among these individuals. Furthermore, there were no differences in ON tendency across BMI groups. Further studies are needed on the physical appearance concerns, behaviors of weight control and weight loss, as well as the diet quality of people with ON.

## Data availability statement

All the data used for the analyses presented within the paper are stored at the open science repository, which is available at the link: https://doi.org/10.18150/RRBUQD.

## Author contributions

MP: Conceptualization, Data curation, Formal analysis, Funding acquisition, Investigation, Methodology, Project administration, Resources, Visualization, Writing – original draft. JK: Writing – review & editing.
