# Supplementary material for: Orthorexic tendency and its association with weight control methods and dietary variety in Polish adults: a cross-sectional study
Source: Front Nutr. 2024 Apr 22;11:1355871. doi: 10.3389/fnut.2024.1355871 (PMC11073497; doi:10.3389/fnut.2024.1355871)
Supplement: Supplementary file 1 [file Image_1.pdf]

*Supplementary Material*

# Orthorexic Tendency and Its Association with Weight Control Methods and Dietary Variety in Polish Adults: A Cross-Sectional Study

**Marta Plichta*** **and Joanna Kowalkowska**

*** Correspondence:** Marta Plichta: marta_plichta@sggw.edu.pl

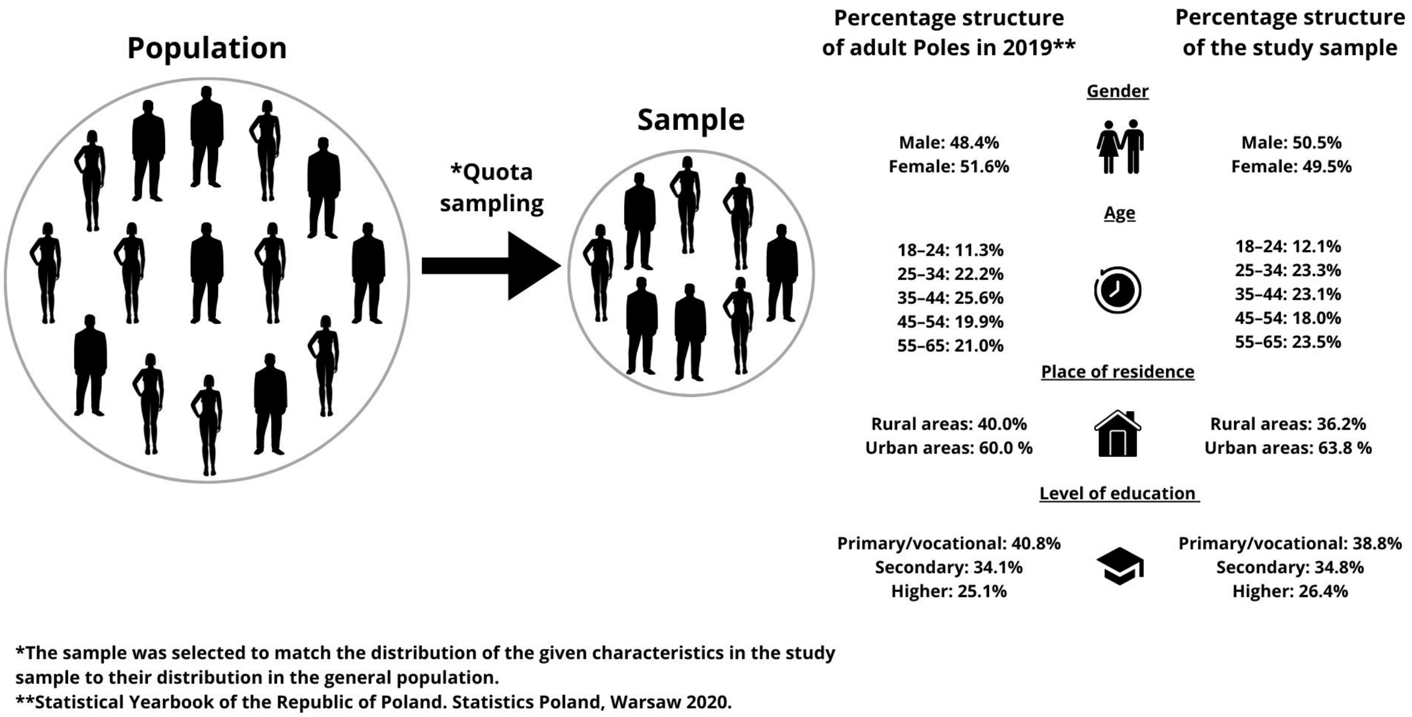


**Supplementary Figure S1.** Quota sampling of adult Poles in the study.
